# Supplementary material for: End Sequence Analysis Toolkit (ESAT) expands the extractable information from single-cell RNA-seq data
Source: Genome Res. 2016 Oct;26(10):1397–410. doi: 10.1101/gr.207902.116 (PMC5052061; doi:10.1101/gr.207902.116)
Supplement: Supplemental Material [file supp_gr.207902.116_Supp_Materials.tar.gz › ESAT-esat_v0.1/doc/html/classbroad_1_1pda_1_1geneexpression_1_1clustering_1_1_pearson_distance.html]

ESAT: broad.pda.geneexpression.clustering.PearsonDistance Class Reference


ESAT


- Main Page
- Related Pages
- Packages
- Classes
- Files

- Class List
- Class Index
- Class Hierarchy
- Class Members

All Classes Namespaces Files Functions Variables Enumerator Pages

- broad
- pda
- geneexpression
- clustering
- PearsonDistance

Public Member Functions |
List of all members

broad.pda.geneexpression.clustering.PearsonDistance Class Reference

Inheritance diagram for broad.pda.geneexpression.clustering.PearsonDistance:

[legend]

Collaboration diagram for broad.pda.geneexpression.clustering.PearsonDistance:

[legend]

|  |  |
| --- | --- |
| Public Member Functions | |
| double | measure (double[] a, double[] b) |
|  | |
| String | getName () |
|  | |

## Member Function Documentation

|  |  |  |  |  |
| --- | --- | --- | --- | --- |
| String broad.pda.geneexpression.clustering.PearsonDistance.getName | ( |  | ) |  |

Implements broad.pda.geneexpression.clustering.ClusterDistanceFunction.

|  |  |  |  |
| --- | --- | --- | --- |
| double broad.pda.geneexpression.clustering.PearsonDistance.measure | ( | double[] | *a*, |
|  |  | double[] | *b* |
|  | ) |  |  |

Implements broad.pda.geneexpression.clustering.ClusterDistanceFunction.

---

The documentation for this class was generated from the following file:

- java/broad/pda/geneexpression/clustering/PearsonDistance.java


---

Generated on Fri Aug 15 2014 15:11:01 for ESAT by  

 1.8.7
